# Bone microarchitectural parameters can detect oxytocin induced changes prior to bone density on mitigating bone deterioration in rabbit osteoporosis model using micro-CT

**DOI:** 10.1186/s12891-019-2861-0

**Published:** 2019-11-23

**Authors:** Yuyou Qiu, Cuisong Tang, Mario Serrano-Sosa, Jian Hu, Jingqi Zhu, Guangyu Tang, Chuan Huang, Mingqian Huang

**Affiliations:** 1 Department of Radiology, Shanghai Tenth People’s Hospital, Tongji University School of Medicine, 301 Middle Yanchang Road, Shanghai, 200072 China; 2 Department of Biomedical Engineering, Stony Brook University, Stony Brook, New York, 11794 USA; 3 Department of Radiology, Stony Brook Medicine, Stony Brook, New York, 11794 USA

**Keywords:** Osteoporosis, X-ray microtomography, Bone marrow, Bone micro-architectural parameters, Oxytocin

## Abstract

**Background:**

This study is aimed to determine the efficacy of X-Ray Microtomography (micro-CT) in predicting oxytocin (OT) treatment response in rabbit osteoporosis(OP) model.

**Methods:**

Sixty-five rabbits were randomly divided into three groups: control group, ovariectomy (OVX) -vehicle and OVX-oxytocin group. The controls underwent sham surgery. OVX-vehicle and OVX-oxytocin groups were subjected to bilateral OVX. The rabbits in OVX-oxytocin group were injected with oxytocin. In the 0th, 4th, 8th, 10th and 12th weeks post OVX operation, bone mineral density (BMD) and bone micro-architectural parameters were measured in three groups.

**Results:**

Bone mineral density (BMD), bone volume fraction (BV/TV), Trabecular Number (Tb.N), and Trabecular Thickness (Tb.Th) decreased, while Trabecular Spacing (Tb.Sp) and Structure Model Index (SMI) increased overtime in all the three groups. In OVX-oxytocin group, the bone deterioration tendency is slowing down compared with that of the OVX-vehicle group. The BMD of the OVX-oxytocin group was significantly lower than those in the OVX-vehicle group at 12th week (*P* = 0.017). BV/TV and Tb.Sp in OVX-oxytocin group changed significantly from 8th week (*P* = 0.043) and 12th week (*P* = 0.014), which is earlier than that of BMD and other bone micro-architectural parameters.

**Conclusion:**

BV/TV and Tb.Sp changed prior to BMD and other bone micro-architectural parameters with oxytocin intervention, which indicate that they are more sensitive markers for predicting early osteoporosis and treatment monitoring when using micro-CT to evaluate osteoporosis rabbit model.

## Background

Osteoporosis (OP) is a metabolic bone disease that affects the whole skeletal system and characterized by general reduced bone density from bone micro-architectural deterioration. According to literature, about 40% of post-menopausal females suffer from osteoporosis globally [1]. People who suffer from osteoporosis commonly encounter fragility fracture thus create a tremendous burden on the society. Therefore, better understanding of the disease process leading to prevention, early diagnosis and treatment is important [2]. Estrogen deprivation of experimental osteoporosis model in animals, such as rats, rabbits, is most commonly used for the postmenopausal osteoporosis study.

There are extensive literatures using X-Ray Microtomography (micro-CT) to study mouse/mice bone micro-architectural changes. It can accurately detect BMD and other micro-structure parametersas to assess early bone degeneration in animal OP models. Bilateral ovariectomy (OVX) rats are generally used as a classical OPanimal model for postmenopausal OP research. However, the rat skeletal system is different from human of lacking Haversian system and limited bone remodeling according to the literature [3]. These shortcomings create a problem for using this model to assess some of the newer osteoporosis treatment models. For example, the bone-forming agents, which generate dynamic trabecular and cortical bone remodeling in combating osteoporosis. The rabbit model is a lot similar to human skeletal system and more appropriate for use [3, 4].

There are several advantages of the rabbit model. First, there is Haversian system in the rabbit bone. Second, the cortical reconstruction activity in rabbit is like humans. On top of that, the rabbit bone transformation and maturation are faster than rats. Therefore, the experimental results of rabbit model with drug intervention for osteoporosis treatment should be theoretically closer to human process than mouse/rat model [5] and more relevant for clinical practice.

Micro-CT has been reported as a reliable high-throughput method to assess fetal skeletons in developmental osteoporosis studies [6–8]. Micro-CT can measure bone mineral density (BMD) and micro-architectural parameters reflecting cancellous bone, such as bone volume fraction (BV/TV), Trabecular Number (Tb.N), Trabecular Thickness (Tb.Th), Trabecular Spacing (Tb.Sp) and Structure Model Index (SMI). Other parameters reflecting cortical bone, such as bone surface-to-volume ration (BS/BV), connectivity density (Conn. N), can also be obtained. BV/TV represents the fraction of a given volume of interest, total volume, or tissue volume that is occupied by bone. It can evaluate relative changes in bone micro volume present from one time point to another or after a given treatment. Tb.Th and Tb.Sp are measures of the 3-D structure of cancellous bone. Tb.N is taken as the inverse of the mean distance between the mid-axes of the structure to be examined. SMI describes the degree to which the trabecular network follows common plate-like or rod-like structural models. It is known that the metabolic turnover rate of cancellous bones is 8 times higher than that of cortical bones. The early stage of osteoporosis is characterized by sparse, thinned and broken trabeculae. In this pilot study, we mainly studied those parameters representing cancellous bone to reflect earlier bone degeneration than BMD after OT intervention. Thus, micro-CT can effectively evaluate both bone quantity and bone quality of osteoporosis at early stage by BMD and microstructural parameters reflecting cancellous bone [9, 10].

As of now, bone restoration treatment has many side effects. Hormone therapy can maintain bone mass and reduce bone marrow fat, but it increases the risk of breast cancer and cardiovascular disease [11].. The current treatments for osteoporosis include anti-absorption therapy, such as bisphosphonate, calcitonin, and selective estrogen receptor modulator [12]. Oxytocin (OT) is an endogenous endocrine hormone. Its receptors are positively regulated by estrogens, through estrogen receptor-β because oral administration of estrogen induces an increase in oxytocin serum levels within 12 h in humans [13]. In-vitro studies have found that oxytocin is one of the major regulators in bone marrow mesenchymal stem cell differentiation, which promotes its differentiation into osteoblasts and inhibits differentiation into adipocytes [13–16]. Experimental research proved that after ovariectomy, mice plasma oxytocin values markedly decreased, while the value of palsma oxytocin decreased in the control group. Plasma oxytocin values of post-menopausal women suffering from osteoporosis were lower than those of healthy post-menopausal women [15, 17]. Beranger et al [28] found OT to be a prominent strategy for treating osteopenia, overweight, and fat mass redistribution without any detrimental effects in a OP mouse model. Wang M et al [29] reported OT promoted peri-implant bone healing and osseointegration of titanium implant and recovered the negative effects of OP in undisturbed bone tissue partially. Butezloff MM [30] reported vibration therapy improved bone quality and the quality of the fracture bone callus in ovariectomized rat.

In this experiment, we use micro-CT to study early in-vivo oxytocin efficacy on mitigating bone deterioration in rabbit osteoporosis model.

## Methods

### Animals and experimental design

**Fig. 1:**
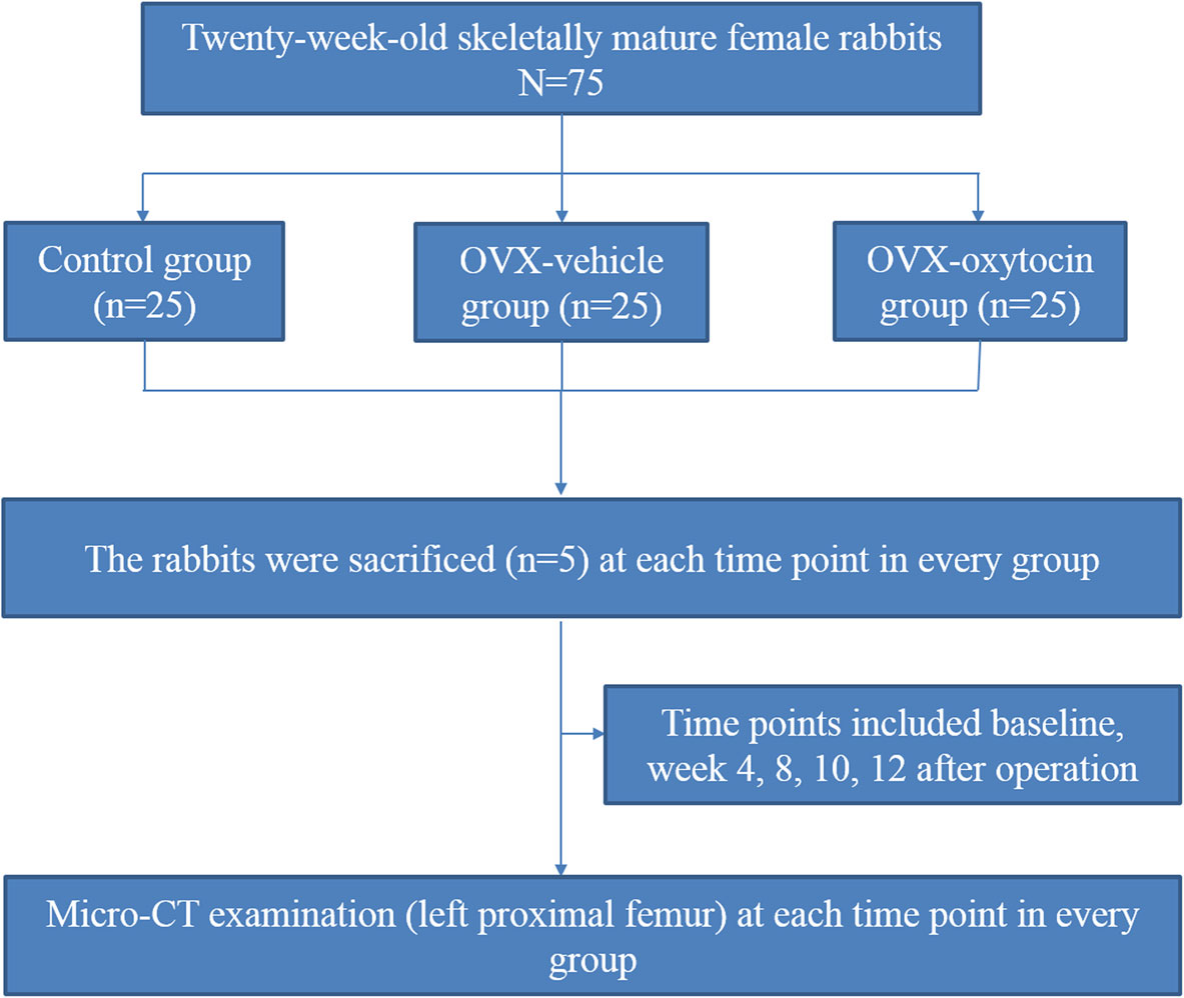
Study flowchart

**Fig. 2:**
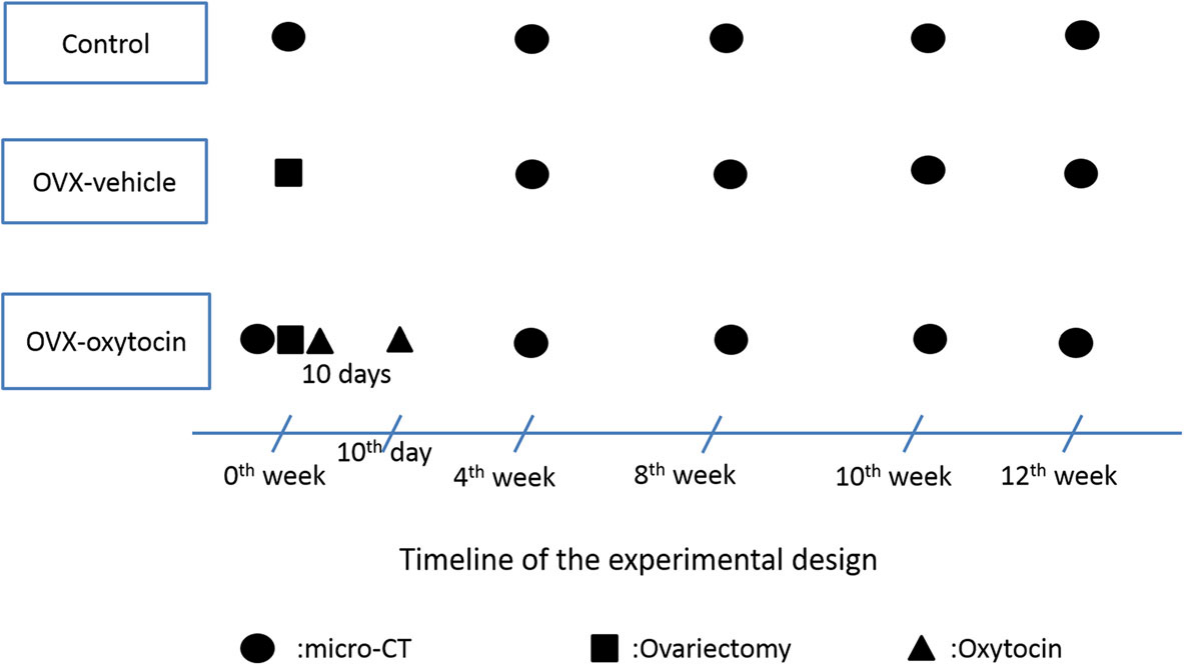
Timeline of experimental design

This experiment was approved by the Ethics Committee of Shanghai Science and Technology Commission [SYXK (Shanghai) 2014–0115] and the animal evaluation committee of Shanghai Tenth People’s Hospital of Tongji University. Animal experiment of our research are strictly followed the nursing guidelines and the experimental animal use system established by the Ministry of Science and Technology of China in 2006. Rabbit bone has a quick remodeling manner with Haversian system. It takes a relatively short time for bone maturity of rabbit of approximately 20 weeks [29]. Therefore, seventy-five 20-week-old (2.6 ± 0.46 kg) New Zealand white rabbits were used in the study (Department of Laboratory Animal Science, Tongji University, Shanghai, China). Five rabbits died throughout the study period, among which 1in the OVX-Vehicle and 1 in OVX-Oxytocin group died from diarrhea respectively, 1 died from anesthesia overdose during operation, 2died from other unknown causes. Rabbits were divided into three groups (*n* = 25 per group): control group; OP group (OVX-vehicle); and OP+ oxytocin group (OVX-oxytocin group). Daily saline injection are performed for 10 consecutive days after sham surgery in the control group. The sham surgery was to hold up the ovaries and then returned to their original position. The OVX-vehicle group was subjected to OVXonly. The OVX-oxytocin group was treated with OVX and subcutaneous injection of 1 mg/kg of oxytocin daily for 10 days after OVX or up to the experimental points [15, 18]. To prevent infection, rabbits all received antibiotics (penicillin 4 × 105 U·kg − 1, i.m.b. i.d.) pre-operative and in first five poste-operative days. All rabbits were kept in their individual cages with 12-h light dark cycle and 20–26 °C degree constant temperature. The rabbits were weighted every week for dosing purpose. The rabbits in three groups were sacrificed by intravenous sodium pentobarbital overdose (50 mg/kg) at 0th, 4th, 8th, 10th at 12^th^week after operation (five rabbits at each time pointevery group). Afterwards, the femur sample was soaked in 0.9% saline solution gauze and frozen at − 20 °C [18]. Figure 1 shows the study flowchart. Figure 2 shows the timeline of experimental design.

### Micro-CT examination

The femur samples were first dehydrated using 70% ethanol for 48 h. Then micro-CT (GE Health Care, Explore Locus SP, Milwaukee, USA) was performed with the sample remain in 70% ethanol in a holder and placed longitudinally. The micro-CT scanning parameters were [19]: Tube voltage, 80 kV; 400 ms per frame of exposure time; 450 uA for current; 0.5 for rotation step, with a 360° complete rotatio; 18 min of acquisition time. The images were reconstructed at isotropic resolution as 45 um × 45 um × 45 um. The images were Gaussian filtered (sigma = 0.8, support = 1 voxel) and binarized using a global thresholding procedure to separate critical bone from trabecular bone [20–23]. The size for ROI is 5 mm (AP) × 6 mm (RL) × 17 mm (FH). The ROI is superiorly placed 1.0 mm from the distal growth plate of the femoral head within the femoral cortex. All images were post-operated to isolate cancellous bone from cortical bone and preserve its morphology using a threshold of 800 manually. Bone mineral density (BMD), structure model index (SMI), bone volume fraction (BV/TV), trabecular thickness (Tb.Th), trabecular number (Tb.N),and trabecular separation (Tb.Sp) parameters were determined [20]. Micro-CT scanning was operated by two professional technicians with 6 years working experiences. Scans were repeated three times, average BMD and bone micro-architectural parameters of each group were taken from the same region of interest (ROI). All the micro-CT raw datas were listed in Additional file 1

### Statistical analysis

SPSS 17.0 statistical analysis software was used. Shapiro–Wilk was used to test normalized distribution of data. Differences in BMD and micro-architectural parameters among the three groups were analyzed using two-way ANOVA and Bonferroni post hoc test. Paired t test was used to compare parameters between two groups at different time points. *P* < 0.05 was considered statistically significant.

## Results

### Determination of BMD and bone micro-structure parameters

**Fig. 3:**
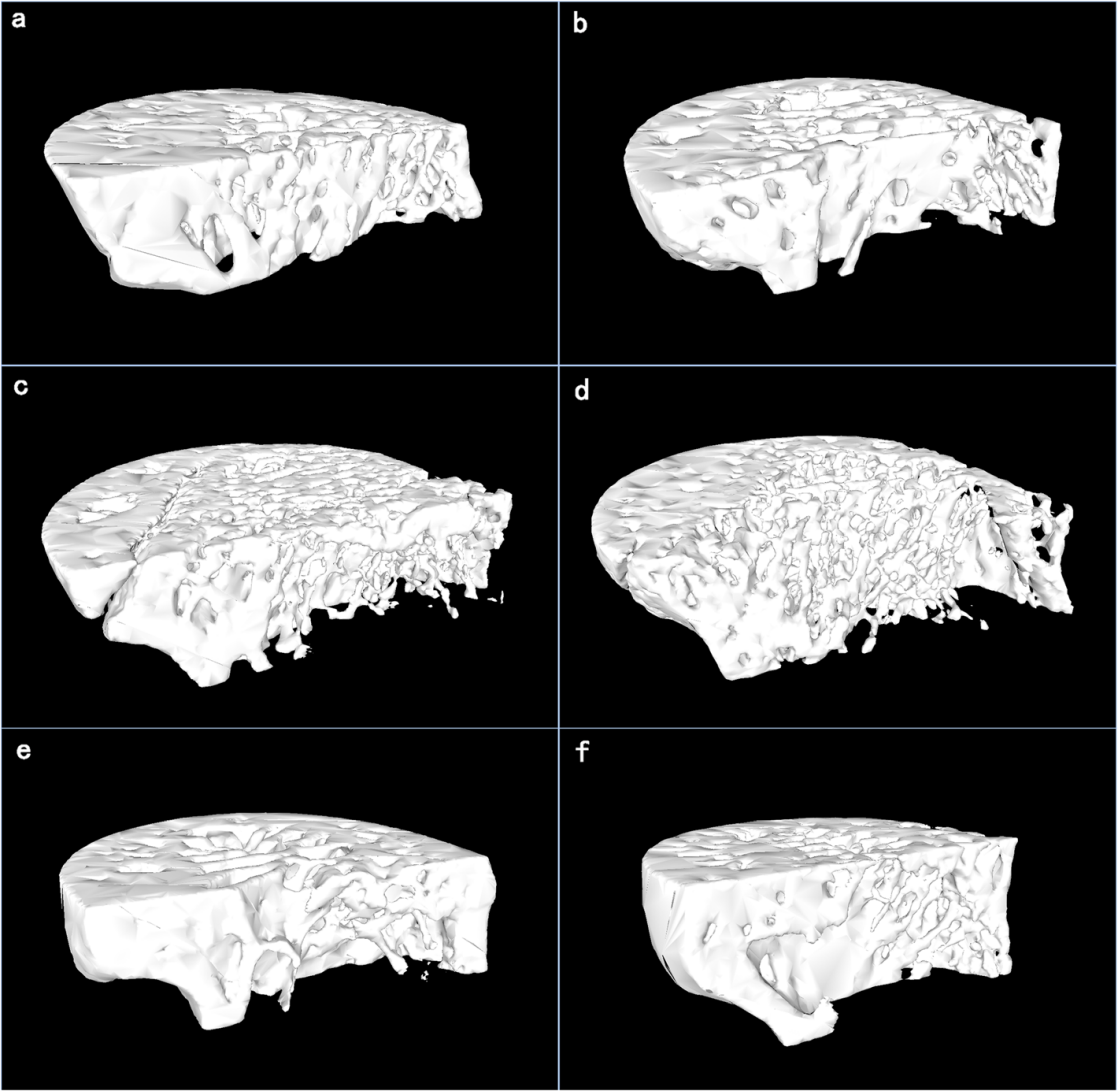
**a-f** 3D micro-CT images of the proximal femurs in the three groups.3.1 3D images of a rabbit in control group at 12th week show that bone loss is relatively slight and the femur trabecular microarchitectural is largely preserved. Subpanels 3–2, 3-3,3–4 represent the 3D images of a rabbit in OVX-vehicle group at 4th, 8th, and 12th week after OVX respectively. The images show continuous cancellous bone microarchitectural deterioration, trabeculae decrease, and enlarged interspace over time. Subpanels 3–5 and 3–6 are the 3D images of a rabbit in OVX-oxytocin group at 4th and 12th week after OVX, respectively. The cancellous bone architecture has significantly been preserved compared with those in OVX-vehicle group of the same time point

The coefficients of variability in this study were as follows: BMD 1.87–2.01%, BV/TV 0.72–0.85%, SMI 0.94–1.27%, Tb.N 0.81–0.96%, Tb.Th 0.69–0.86%, Tb.Sp 0.88–1.07%. Table 1 shows determination results of bone micro-structural parameters of specimens on upper segment of left thigh bone of three groups of experimental rabbits at representative time points. Figure 2a-f shows longitudinal changes of BMD and bone micro-architectural parameters of specimens taken from the upper segment of the left femur of 3 groups of experimental rabbits at consecutive time points (from 0th to 12th week). Figure 3a-f shows detailed 3D images of the upper segment of left femur at same representative time points. Three Intergroup comparisons have been performed between control, OVX-vehicle group and OVX-oxytocin group. *P* values presented are not corrected for multiple comparisons.

**Table 1:** Micro-CT analysis of femora trabecular parameters of proximal femur in three groups at different timepoint after operation

Time point	BMD (mg/cm^2^)			SMI		
	control	OVX-vehicle group	OVX-oxytocin group	control	OVX-vehicle group	OVX-oxytocin group
baseline	102.73 [89.98,115.48	98.57 [83.58,113.56]^☆^	97.58 [81.33,113.83]^☆^	0.67 [0.55,0.79]	0.69 [0.46,0.92]^☆^	0.67 [0.52,0.82]^☆^
4^th^w	91.52 [78.67,104.37]	93.68 [84.94,102.42]	95.75 [82.47,109.03]	0.70 [0.50,0.90]	0.97 [0.80,1.14]	0.71 [0.59,0.83]
8^th^w	82.05 [68.83,95.27]	57.18 [41.36,73.00]^*^	74.66 [60.09,89.23]^#^	0.75 [0.58,0.92]	1.17 [0.97,1.37]^*^	0.75 [0.47,1.03]^#^
10^th^w	65.24 [56.68,73.80]	35.32 [27.18,43.46]^*^	58.65 [42.10,75.20]^#^	0.78 [0.58,0.98]	1.27 [1.01,1.53]^*^	0.79 [0.64,0.94]^#^
12^th^w	59.11 [46.76,71.46]	24.47 [8.71,40.23]^*^	50.58 [40.95,60.21]^*#^	0.79 [0.65,0.93]	1.45 [1.33,1.57]^*^	0.86 [0.72,1.00]^*#^
Time point	BV/TV (%)			Tb.N (mm^−1^)		
	control	OVX-vehicle group	OVX-oxytocin group	control	OVX-vehicle group	OVX-oxytocin group
baseline	41.25 [39.57,42.93]	40.14 [38.13,42.15]^☆^	41.36 [38.86,43.86]^☆^	2.71 [2.39,3.03]	2.68 [2.42,2.94]^☆^	2.71 [2.59,2.83]^☆^
4^th^w	38.70 [35.95,41.45]	36.63 [34.32,38.94]	38.28 [37.34,39.22]	2.64 [2.39,2.89]	2.34 [2.15,2.53]	2.58 [2.34,2.82]
8^th^w	38.05 [34.96,41.14]	33.69 [31.21,36.17]^*^	37.48 [36.24,38.72]^#^	2.61 [2.50,2.72]	2.29 [2.14,2.44]^*^	2.52 [2.20,2.84]^#^
10^th^w	37.54 [35.97,39.11]	28.57 [27.37,29.77]^*^	36.91 [34.85,38.97]^#^	2.53 [2.25,2.81]	2.24 [2.09,2.39]^*^	2.47 [2.29,2.65]^#^
12^th^w	36.51 [35.27,37.75]	27.44 [25.11,29.77]^*^	33.58 [31.93,35.23]^*#^	2.49 [2.33,2.65]	2.06 [1.84,2.28]^*^	2.30 [2.08,2.52]^*#^
Time point	Tb.Th (μm)				Tb.Sp (μm)	
	control	OVX-vehicle group	OVX-oxytocin group	control	OVX-vehicle group	OVX-oxytocin group
baseline	171.56 [164.59,178.53]	169.24 [160.43,178.05]^☆^	172.34 [162.10,182.58]^☆^	203.75 [194.08,213.42]	204.17 [194.92,213.42]^☆^	203.41 [194.67,212.15]^☆^
4^th^w	167.80 [156.60,179.04]	163.02 [148.52,177.52]	168.36 [156.19,180.53]	205.20 [200.02,210.38]	228.34 [217.69,238.99]	207.11 [197.16,217.06]
8^th^w	165.24 [154.40,176.08]	156.68 [145.48,167.88]	162.08 [153.84,170.32]	208.36 [200.62,216.10]	256.14 [247.94,264.34]^*^	217.04 [209.82,224.26]^#^
10^th^w	162.47 [151.24,173.70]	136.54 [127.40,145.68]^*^	159.87 [151.99,167.75]^#^	209.89 [201.75,218.03]	279.34 [263.87,294.81]^*^	230.53 [222.76,238.30]^#^
12^th^w	163.40 [150.86,175.94]	133.28 [124.61,141.95]^*^	152.20 [141.87,162.53]^*#^	216.47 [207.19,225.75]	319.08 [307.94,330.22]^*^	259.66 [251.43,267.89]^*#^

*BMD* Bone mineral density, *BV/TV* Bone volume fraction, *SMI* Structure model index, *Tb.N* Trabecular plate number, *Tb.Th* Trabecular thickness, *Tb.Sp* Trabecular spacing, ^*^P < 0.05 comparison with the control group at different time points; ^#^*P* < 0.05 comparison with OP group at different time points; ^☆^*P*>0.05 comparison with base point (0 week) at different time points

As shown in Fig. 2a-f the experimental results of three groups have a common trend of bone deterioration. BMD, BV/TV, Tb.N, Tb.Th all decreased during the study, while Tb.Sp and SMI increased overtime in all three groups. Most strikingly, in OVX-oxytocin group, the bone deterioration tendency is slowed down compared with that of the OVX-vehicle group, for all parameters. More interestingly, bone micro-architectural parameters BV/TV and Tb.Sp are statistically different between OVX-oxytocin group and OVX-vehicle group from 8th week on, which is early than other micro-architectural parameters and BMD.

As shown in Fig. 2a, in the control group, compared with baseline (0th week), BMD demonstrated a gradually descending trend. BV/TV, Tb.N and Tb.Th gradually decreased from 0th week to 12th week, while Tb.Sp and SMI increased overtime. In comparison to 0th week, BMD value in the control group decreased by 30.3% (*P* = 0.024) at 8th week, 45.9% (*P* < 0.01) at 10th week and 58.6% (*P* < 0.01) at 12th week.In OVX-vehicle group, BMD, BV/TV, Tb.N, and Tb.Th significantly decreased after OVX operation from 4th week, meanwhile SMI and Tb.Sp significantly increased from 4th week on,comparedto the control group.

In OVX-oxytocin group, BMD were not significantly different until 12th week after operation compared toOVX-vehicle group (*P* = 0.017). Tb.N and Tb.Th values increased by 25.21% (*P* = 0.028) and 37.09% (*P* = 0.017) at 10th week, compared to those of OVX-vehicle group. Meanwhile, SMI values in OVX-oxytocin group, decreased by 18.47% (*P* = 0.041) at the 10th week and 54.47% (*P* < 0.01) at the 12th week, compared to those of OVX-vehicle group. Notably, from 8th week on, the BV/TV of OVX-oxytocin group had a significant increase of 17.25% (*P* = 0.043), 49.19% (*P* < 0.01), and 21.37% (*P* = 0.031) at 8th,10th and 12th week, respectively, compared to those of OVX-vehicle group. Tb.Sp decreased by 44.48% (*P* = 0.014), 58.67% (*P* < 0.01) and 71.23% (*P* < 0.01)at 8th,10th and 12th week, respectively,when compared to those of OVX-vehicle group. Both BV/TV and Tb.Sp changed earlier after oxytocin intervention than that of BMD, Tb.N, Tb.Th and SMI.

Our results showed that BMD had a consistent descending trend along with bone micro-architectural parameters such as BV/TV, Tb.N, Tb.Th, whereas Tb.Sp and SMI increased overtime in rabbit osteoporosis model. Early OT injection in OVX-oxytocin could effectively mitigate bone deterioration compared with that of control and OVX-vehicle group. Both BV/TV and Tb.Sp changed earlierafter oxytocininterventionthan that of BMD, Tb.N, Tb.Th and SMI.

### Weights of experimental rabbits

The body weight in the OVX-oxytocin group decreased slightly, but no significant differences in body mass were observed when compared with that at baseline or with the data at the same time point with other two groups (OVX-oxytocin group versus control group, *P* = 0.733, 0.248, 0.624, 0.673, 0.071 from baseline to 12th week respectively. OVX-oxytocin group versus OVX-vehicle group, *P* = 0.528, 0.174, 0.492, 0.616, 0.259 from baseline to 12th week respectively).

## Discussion

We used a compact micro-CT scanner to monitor the micro-architectural changes in the rabbit’s cancellous bone over time. Our main goal is to find which parameter of micro-CT examination is a more sensitive and effective marker as to predict early bone degeneration.The mechanism of how OT mitigate bone deterioration had been explored by magnetic resonance spectroscopy and pathological examination in our previous publications [18]. Therefore, the results of other imaging techniques (DXA, MRS, etc.) or blood plasma biomarkers were not performed in this experiment. In our study, we studied three groups of rabbits including: a control (sham-operated) group, an ovariectomized group (OVX-vehicle) and an ovariectomized group with oxytocin treatment started 2 weeks after the surgery. CT measurements were taken consistently for 12 weeks after the surgery of all three groups. Different pattern of changes in rabbit cancellous bone micro-architecture were observed between the three groups over time. Micro-CT imaging was performed to quantitatively measure the bone mass on the basis of BMD and most common micro-architectural parametersof cancellous bone including: BV/TV, Tb.N, Tb.Th, Tb.Sp, SMI, which reflect bone micro volume, trabecular bone number, thickness, spacing, and trabecular network, respectively [8]. Our study found that BV/TV and Tb.Sp changed prior to BMD and other bone micro-architectural parameters with oxytocin intervention, which indicate that they are more sensitive markers for predicting early osteoporosis and treatment monitoringwhen using micro-CT to evaluate osteoporosis rabbit model.

Boyd et al [5] reported rat had a 33% of bone volume decreases 1 month after OVX, and 72% after 3 months compared to baseline measurements. Significant changes in bone volume ratio, trabecular number and separation were found. Perillie E et al [19] used micro-CT to study the morphometric changes in the tibiae of the rats. They studied three groups of rats including a group of ovariectomized rats, a group of sham-operated rats and a group of ovariectomized rats with zoledronic acid treatment. The study demonstrated a decrease in BV/TV, Tb.N and increase in Tp.Sp of the OVX group with most rapid change happened between week 4 and 8. For the group receiving zoledronic treatment, BV/TV was similar to the baseline at week 4. However, during the first 2 weeks after surgery of ovariectomy, there was increase in Tb.Sp and SMI with decrease in Tb.N, signaling bone loss. After 2 weeks of treatment with zoledronic acid, there appears to be reversing course of bone loss.Allison R. Altman et al [21] used the micro-CT to study young and adult rat tibiae with sham operation or intermittent parathyroid hormone treatment. The new bone of the parathyroid hormone-treated rats had greater trabecular number, trabecular bone volume fraction, and trabecular thickness than those of the sham operation rats, demonstrating the PTH’s anabolic effect on bone modeling.

We observed decreases in BV/TV, Tb.N, and Tb.Th, and increase in SMI in the control (sham operation) group. These changes indicate bone mass loss as in aging. This result is consistent with literature of reported findings in both rats and rabbits with bone growth [18, 22, 23]. Continuous deterioration of cancellous bone was observed of the ovariectomized rabbits (OVX-vehicle group). There were decrease in BMD, BV/TV, Tb.N, Tb.Th and increase in Tp.Sp and SMI. The change first appeared 4th week post operatively. And by 8th post-operative week, the changes plateaued. These findings were also in agreement with literatures [18, 24]. Oxytocin injection acts through preventing the cancellous bones decrease in BV/TV and expansion of trabecular bone spacing. From 8th week on after ovariectomy, the OVX-oxytocin group BV/TV was significantly higher than that of OVX-vehicle group at 8th(17.25%, *p* = 0.043), 10th (49.19%, *p* < 0.01) and 12th week (21.37%, *p* = 0.031). Additionally, Tb.Sp decreased by 44.48% (*p* = 0.014), 58.67% (*p* < 0.01) and 71.23% (*p* < 0.01) respectively from 8th to 12th week, when compared with those of OVX-Vehicle group.Thus, we demonstrated that early oxytocin treatment prevented cancellous bone loss secondary to the surgery of OVX. Additionally, the structural parameters returned to baseline. At 8th week, significant increase in BV/TV and decrease in Tb.Sp were observed of the OVX-oxytocin group.This change suggests that there is bone growth in OVX-oxytocin group, while OVX-vehicle group showed continuous bone loss. These findings indicate BV/TV and Tb.Sp could be more sensitive indexes than BMD or other bone micro-architectural parameters in predicting oxytocin intervention efficacy for osteoporosis treatment.

In our experiment, bone substances of rabbits in control group, OVX-vehicle group and OVX-oxytocin group at initial stage (0th week) were kept relatively stable, and afterwards, marked bone deterioration appeared in different time points varied within three groups. After OVX operation, bone micro-architectural parameters BV/TV, Tb.N and Tb.Th rapidly decreased, meanwhile Tb.Sp and SMI rapidly increased, which indicated that osteoporosis evolving process was accompanied by microscopic changes like attenuation, puncturing and fracture of bone trabecula until micro-fracture happened and connecting structure was damaged, which were manifested by small and sparse bones, osteopenia, broadened trabecular space, transition of bone trabecula from plate shape to rod shape and increased SMI. We observed that bone loss has been largely preserved in rabbit osteoporosis model with oxytocin intervention. This result is in accordance with drug treatment response of osteoporosis rat model in the literature [9, 21]. Moreover, micro-architectural parameter of BV/TV significantly decreased while Tb.Sp significantly decreased from 8th week on in OVX-oxytocin group, which changed prior to BMD, Tb.N, Tb.Th and SMI. Thus, our experiment results showed that bone micro-architectural parameters, BV/TV and Tb.Sp are more efficient parameters than BMD in predicting the mitigation of oxytocin treatment in rabbit osteoporosis model.

One of the strengths of our study is the use of rabbit OVX model.Rabbits have Haversian system that enable them to remodel and achieve skeletal maturity quickly. Rabbits become sexually mature around 20 to 24 weeks of age and usually reach skeletal maturity around 38 to 32 weeks [18]. By the age of 32–36 weeks, rabbits usually have reached peak bone mass with regular bone resorption and formation. Therefore, the changes of bone tissues can be observed within a short term [25]. The OVX operation combined with intramuscular cortical hormones injection of New Zealand white rabbits done by Casfieda et al [3] was successful to induce osteoporosis in a short time. Thus, provided a great model for longitudinal imaging evaluation of osteoporosis research due to its simple approach and low cost.

Oxytocin is a potential high-efficiency therapy for treating osteoporosis. The main physiological function of oxytocin is to boost uterine contraction in delivery period and milk ejection in lactation period, and it is sustainably secreted in human body for the whole life and influenced from ovarian hormone level and age increasing factors [26, 27]. It has been found that oxytocin has direct regulating effect on bonemetabolism and it has been verified that oxytocin participates in regulating bone remodeling of the parent in gestation period, and oxytocin receptors exist on surfaces of both osteoblasts and osteoclasts [27]. Elabd et al. [15] proved that oxytocin is one of the main factors regulating osteogenic/adipogenetic differentiation of mesenchymal stem cells. Through oxytocin intervention of pluripotent adipose differentiation stem cells and bone marrow stem cells, it’s found that oxytocin and its consubstantial matters could facilitate osteogenic differentiation of stem cells and inhibit its adipogenesis, which revealed that oxytocin played a significant role in osteogenic/adipogenetic differentiation process of mesenchymal stem cells. Beranger et al [28] reported that after ovariectomy, oxytocin injection was implemented for consecutive 2 weeks when bone metabolism and absorption decreased, or oxytocin was injected when the degree of osteoblasts and osteoclasts activities decreased 8 weeks after ovariectomy operation, and both had the effects of inhibiting bone deterioration. In-vivo studies also found that oxytocin has a role in inhibiting bone decay in rat osteoporosis model [10]. Therefore, oxytocin is a potential alternative therapy for treating osteoporosis.

Our study has several limitations. First, the number of rabbits that were included in our study were relatively small at various time points, which could potentially increase experimental errors from individual differences. Secondly, side effects from oxytocin injection such as lowered free activity of rabbit, food intake changes and breathing/aeration ratio were not recorded. Even though, we did not observe any change in food intake and metal condition of the rabbits in all three groups. Thirdly, we did not record the changes ofbone biomarkersin blood plasma,such as estrogen, alkaline phosphatase, Tartaric acid phosphatase, etc. So, the interplay between plasma biomarkers, bone mineral density and bone micro-architecture could not be studied in this experiment. Lastly, the dose of oxytocin we used in the adult rabbit was different from what would be used in humans for treating osteoporosis. Further study with clinical model that can be applied to human would be useful.

## Conclusions

In summary, longitudinal micro-CT imaging was performed and its efficacy for distinguishing the skeletal response to oxytocin intervention was showcased in this study. Bone BMD values had the consistent descending trend with bone micro-architectural parameters in rabbit osteoporosis model. Early in vivo oxytocin treatment could effectively mitigate bone deterioration in osteoporosis rabbit model. BV/TV and Tb.Sp changed prior to BMD and other bone micro-architectural parameters with oxytocin intervention, which indicate that they are more sensitive markers for predicting early osteoporosis and treatment when using micro-CT to evaluate osteoporosis rabbit model.

## Supplementary information

**Additional file 1.** Micro-CT raw data.

## Data Availability

All relevant data are included in this manuscript. Additional data may be requested by contacting the corresponding author.

## Abbreviations

BMD: Bone mineral density
BV/TV: Bone volume fraction
micro-CT: X-ray microtomography
OP: Osteoporosis
OT: Oxytocin
OVX: Ovariectomy
ROI: Region of interest
SMI: Structure model index
Tb.N: Trabecular number
Tb.Sp: Trabecular spacing
Tb.Th: Trabecular thickness

## References

[1] Rachner TD, Khosla S, Hofbauer LC (2011). Osteoporosis: now and the future. Lancet.

[2] Compston J (2010). Osteoporosis. Social and economic impact. Radiol Clin N Am.

[3] Castaneda S, Calvo E, Largo R, Gonzalez-Gonzalez R (2008). Characterization of a new experimental model of osteoporosis in rabbits. J Bone Miner Metab.

[4] Brouwers JE, Lambers FM, Gasser JA, van Rietbergen B, Huiskes R (2008). Bone degeneration and recovery after early and late bisphosphonate treatment of ovariectomized wistar rats assessed by in vivo micro-computed tomography. Calcif Tissue Int.

[5] Boyd SK, Davison P, Muller R, Gasser JA (2006). Monitoring individual morphological changes over time in ovariectomized rats by in vivo micro-computed tomography. Bone.

[6] Waarsing JH, Day JS, Verhaar JA, Ederveen AG, Weinans H (2006). Bone loss dynamics result in trabecular alignment in aging and ovariectomized rats. J Orthop Res.

[7] French J, Gingles N, Stewart J, Woodhouse N (2010). Use of magnetic resonance imaging (MRI) and micro-computed tomography (micro-CT) in the morphological examination of rat and rabbit fetuses from embryo-fetal development studies. Reprod Toxicol.

[8] Winkelmann CT, Wise LD (2009). High-throughput micro-computed tomography imaging as a method to evaluate rat and rabbit fetal skeleta l abnormalities for developmental toxicity studies. J Pharmacol Toxicol Methods.

[9] Bauer NB, Khassawna TE, Goldmann F (2015). Characterization of bone turnover and energy metabolism in a rat model of primary and secondary osteoporosis. Exp Toxicol Pathol.

[10] Brandi ML (2009). Microarchitecture, the key to bone quality. Rheumatology (Oxford).

[11] Chen J, Yu J, He Q (2015). A novel injectable porous surface modified bioactive bone cement for vertebroplasty: an in vivo biomechanical and osteogenic study in a rabbit osteoporosis model. Am J Transl Res.

[12] Jones JR, Barrick C, Kim KA (2005). Deletion of PPARgamma in adipose tissues of mice protects against high fat diet-induced obesity and insulin resistance. Proc Natl Acad Sci U S A.

[13] Choleris E, Gustafsson JA, Korach KS, Muglia LJ, Pfaff DW, Ogawa S (2003). An estrogen-dependent four-gene micronet regulating social recognition: a study with oxytocin and estrogen receptor-alpha and -beta knockout mice. Proc Natl Acad Sci U S A.

[14] Breuil V, Amri EZ, Panaia-Ferrari P (2011). Oxytocin and bone remodelling: relationships with neuropituitary hormones, bone status and body composition. Joint Bone Spine.

[15] Elabd C, Basillais A, Beaupied H (2008). Oxytocin controls differentiation of human mesenchymal stem cells and reverses osteoporosis. Stem Cells.

[16] Tamma R, Colaianni G, Zhu LL (2009). Oxytocin is an anabolic bone hormone. Proc Natl Acad Sci U S A.

[17] Colli VC, Okamoto R, Spritzer PM, Dornelles RC (2012). Oxytocin promotes bone formation during the alveolar healing process in old acyclic female rats. Arch Oral Biol.

[18] Qiu Y, Yao J, Wu X (2015). Longitudinal assessment of oxytocin efficacy on bone and bone marrow fat masses in a rabbit osteoporosis model through 3.0-T magnetic resonance spectroscopy and micro-CT. Osteoporos Int.

[19] Perilli E, Le V, Ma B, Salmon P, Reynolds K, Fazzalari NL (2010). Detecting early bone changes using in vivo micro-CT in ovariectomized, zoledronic acid-treated, and sham-operated rats. Osteoporos Int.

[20] Buie HR, Campbell GM, Klinck RJ, MacNeil JA, Boyd SK (2007). Automatic segmentation of cortical and trabecular compartments based on a dual threshold technique for in vivo micro-CT bone analysis. Bone.

[21] Altman AR, Tseng W, de Bakker CMJ (2015). Quantification of skeletal growth, modeling, and remodeling by in vivo micro computed tomography. BONE.

[22] Hornby SB, Evans GP, Hornby SL, Pataki A, Glatt M, Green JR (2003). Long-term zoledronic acid treatment increases bone structure and mechanical strength of long bones of ovariectomized adult rats. Calcif Tissue Int.

[23] Leitner MM, Tami AE, Montavon PM, Ito K (2008). Longitudinal as well as age-matched assessments of bone changes in the mature ovariectomized rat model. Lab Anim.

[24] Zhu J, Zhang L, Wu X (2017). Reduction of longitudinal vertebral blood perfusion and its likely causes: a quantitative dynamic contrast-enhanced MR imaging study of a rat osteoporosis model. Radiology.

[25] Baofeng L, Zhi Y, Bei C, Guolin M, Qingshui Y, Jian L (2010). Characterization of a rabbit osteoporosis model induced by ovariectomy and glucocorticoid. Acta Orthop.

[26] Yeung DKW, Griffith JF, Antonio GE, Lee FKH, Woo J, Leung PC (2005). Osteoporosis is associated with increased marrow fat content and decreased marrow fat unsaturation: a proton MR spectroscopy study. J Magn Reson Imaging.

[27] Copland JA, Ives KL, Simmons DJ, Soloff MS (1999). Functional oxytocin receptors discovered in human osteoblasts. Endocrinology.

[28] Beranger GE, Pisani DF, Castel J (2014). Oxytocin reverses Ovariectomy-induced osteopenia and body fat gain. Endocrinology.

[29] Wang M, Lan L, Li T (2016). The effect of oxytocin on osseointegration of titanium implant in ovariectomized rats. Connect Tissue Res.

[30] Butezloff MM, Zamarioli A, Leoni GB (2015). Whole-body vibration improves fracture healing and bone quality in rats with ovariectomy-induced osteoporosis. Acta Cir Bras.

